# Pulmonary vein stenoses are reversible early after plumonary vein isolation in patients with paroxysmal atrial fibrillation – a cardiac mri analysis

**DOI:** 10.1186/1532-429X-11-S1-P30

**Published:** 2009-01-28

**Authors:** Christian Mahnkopf, Guido Ritscher, Nathan Burgon, Troy J Badger, Martin Schmidt, Harald Marschang, Klaus Gutleben, Edward DiBella, Nassir F Marrouche, Johannes Brachmann, Anil M Sinha

**Affiliations:** 1 Klinikum Coburg, Coburg, Germany; 2 University of Utah School of Medicine, Salt Lake City, UT USA; 3 Utah Center for Advanced Imaging Research, Salt Lake City, UT USA

**Keywords:** Pleural Effusion, Pulmonary Vein, Cardiac Magnetic Resonance, Maximum Intensity Projection, Cardiac Magnetic Resonance Tomography

## Purpose

To evaluate pulmonary vein diameter and blood flow in patients with PVI using CMR.

## Background

Pulmonary vein isolation (PVI) has become a efficient therapie in patients with paroxysmal atrial fibrillation (AF). A serious complication of PVI is the pulmonary vein stenosis, which is mostly diagnosed several weeks after intervention. The cardiac magnetic resonance tomography (CMR) allows analysis of heart function and pulmonary veins after ablation therapy.

## Methods

Patients with paroxysmal AF were scheduled for CMR (Siemens Espree 1.5 T, Siemens, Germany) before, immediately after, and 24 hours after PVI. For pulmonary vein diameter and blood flow analysis, angiography of the pulmonary veins and flow measurements of the right inferior pulmonary vein (RIPV) were performed. Maximum intensity projections (MIP) were created for offline analysis.

## Results

**Figure 1:**
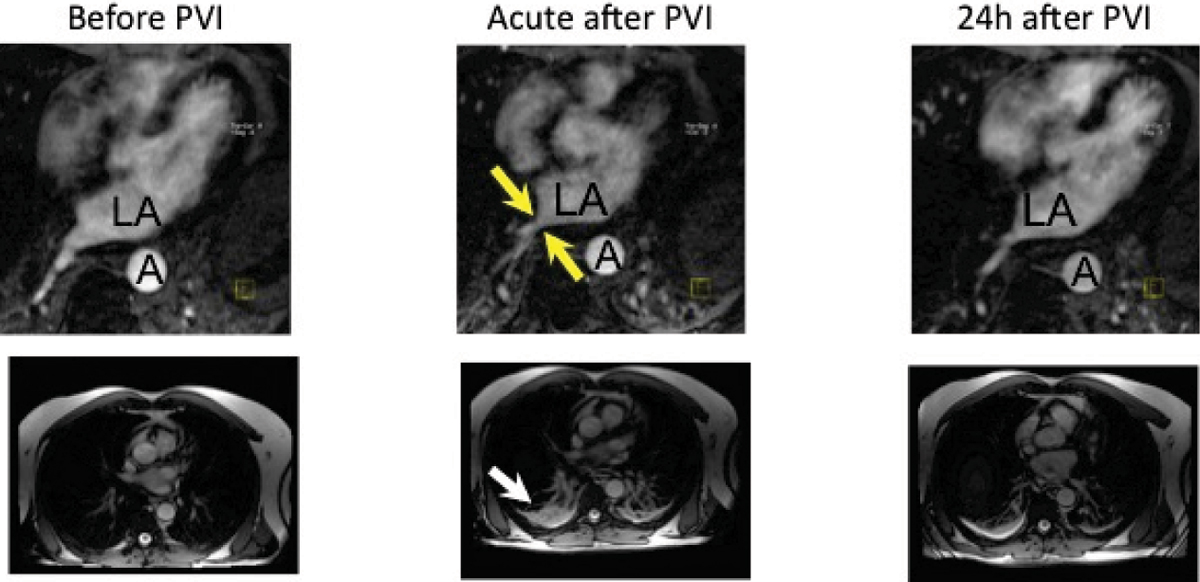
**LA: left atrium; A: aorta**.

15 patients (9 male, 63 ± 9 years) were included in the study. RIPV diameter signifcantly decreased (11.7 ± 1.8 mm vs 9.1 ± 2.4 mm, -22,2%, p < 0.05) during acute, and increased (11.2 ± 2.7 mm, +18,8%, p < 0.05) during 24 h measurements as compared to acute values. RIPV blood flow significantly increased (34.3 ± 14.0 vs. 42.5 ± 14.0 cm/s; +35%; p < 0.05) during acute, and slightly decreased (40.7 ± 13.9; p = n.s.) during 24 h measurements. Also, pulmonary congestion and pleural effusion, which occurred acutely after PVI, were recurrent after 24 h. Figure 1 shows an example of CMR pre, acutely, and 24 h post PVI; arrows indicate pleural effusion (lower row), or RIPV stenosis (upper row).

## Summary

Patients with paroxysmal atrial fibrillation might suffer from pulmonary vein stenosis, pulmonary congestion and pleural effusion early after PVI. As symptoms were regressive within 24 hours after ablation, edema of the vessel tissue might be one the main reasons for the deterioration. Further studies are needed to specify these findings.

